# Efficacy and safety of conscious/deep sedation versus general anesthesia in pulsed field ablation for atrial fibrillation: a systematic review and meta-analysis

**DOI:** 10.3389/fcvm.2026.1933701

**Published:** 2026-10-23

**Authors:** Weiwei Sun, Ruiting Feng, Xiaohong Fu, Nan Zhang, Meng Sun, Min Guo, Jia Gao, Rui Wang

**Affiliations:** 1 The First Clinical Medical College, Shanxi Medical University, Taiyuan, Shanxi, China; 2 Department of Cardiology, First Hospital of Shanxi Medical University, Taiyuan, Shanxi, China

**Keywords:** atrial fibrillation, conscious sedation, deep sedation, general anesthesia, meta-analysis, pulsed field ablation

## Abstract

**Background:**

Pulsed field ablation (PFA) has emerged as a promising therapy for atrial fibrillation. While general anesthesia (GA) is the standard to ensure catheter stability, using conscious or deep sedation (CS/DS) to streamline workflows is gaining interest. However, whether CS/DS provides comparable safety and efficacy to GA remains controversial and has lacked systematic evaluation.

**Objectives:**

To compare the clinical efficacy and safety of PFA for atrial fibrillation under GA vs. CS/DS.

**Methods:**

A literature search was conducted in PubMed, Cochrane Library, and Embase from inception to April 9, 2026. Studies were selected using predefined inclusion and exclusion criteria. The comparison of GA and CS/DS during PFA focused on the following clinical outcomes: procedural time, left atrial dwell time, fluoroscopy time, pulmonary vein isolation (PVI) success rate, and adverse event incidence. Heterogeneity was assessed with the I² statistic and Cochran's *Q* test; sensitivity analysis was performed if heterogeneity was high. Publication bias was evaluated using funnel plots and Egger's test.

**Results:**

Ten studies involving 3,692 atrial fibrillation patients were included. Compared to GA, CS/DS significantly shortened overall procedural time (WMD: −7.73 min, random-effects; 95% CI: −14.17 to −1.29; *P* = 0.019). However, there were no significant differences between the groups in left atrial dwell time (WMD: −2.59 min, random-effects; 95% CI: −9.74 to 4.57), fluoroscopy time (WMD: −2.52 min, random-effects; 95% CI: −5.31 to 0.28), acute PVI success (RR: 1.00, fixed-effects; 95% CI: 0.98 to 1.03), or major adverse events (RR: 1.57, fixed-effects; 95% CI: 0.81 to 3.05). CS/DS showed a borderline trend toward fewer minor adverse events (RR: 0.61, fixed-effects; 95% CI: 0.37 to 1.01; *P* = 0.053).

**Conclusion:**

In PFA for AF, CS/DS was associated with reduced overall procedural time compared with GA, while maintaining comparable acute PVI success rates and safety profiles. However, large-scale randomized controlled trials are still needed to confirm these findings.

**Systematic Review Registration:**

https://www.crd.york.ac.uk/PROSPERO/view/CRD420251165071, identifier (CRD420251165071).

## 1 Introduction

Atrial fibrillation (AF) is the most common cardiac arrhythmia (1). Catheter ablation outperforms antiarrhythmic drugs (2, 3), but conventional thermal modalities—radiofrequency and cryoablation—carry the risk of collateral injury to the esophagus and phrenic nerve. By leveraging unique non-thermal and tissue-selective properties, pulsed field ablation (PFA) precisely targets arrhythmogenic foci while fundamentally minimizing these secondary complications (4, 5). Consequently, PFA has rapidly emerged as a highly promising strategy for AF rhythm control (6).

Optimizing the PFA peri-procedural workflow is a growing clinical priority. High-voltage pulses cause skeletal muscle contractions and pain, posing anesthetic challenges. PFA is currently performed under general anesthesia (GA) or conscious/deep sedation (CS/DS) (7, 8). GA prevents movement and ensures full analgesia (9), but requires mechanical ventilation and specialized staff—increasing resource use and complexity. CS/DS is less invasive and enables faster recovery, offering advantages in resource-limited settings (10). However, sedation may not fully suppress PFA-induced pain or coughing (11), raising a key question: do the logistical benefits of CS/DS compromise procedural safety and acute ablation success?

Although recent studies have explored CS/DS (12–15), small sample sizes and inconsistent findings leave its safety and efficacy relative to GA unresolved. Therefore, we conducted this meta-analysis to compare the clinical outcomes of GA vs. CS/DS in patients undergoing PFA for atrial fibrillation.

## 2 Materials and methods

This study was conducted in accordance with the Preferred Reporting Items for Systematic Reviews and Meta-Analyses (PRISMA) statement, following a prespecified protocol registered in PROSPERO (CRD420251165071). This research received no funding, and the authors declare no financial conflicts of interest.

### 2.1 Literature search strategy

Two researchers (SW and RT) independently designed the search strategy and reached consensus on the inclusion criteria. A systematic literature search was performed in PubMed, Cochrane Library, and Embase from inception to April 9, 2026. Key search terms included “pulsed field ablation,” “atrial fibrillation,” “general anesthesia,” “deep sedation,” “conscious sedation,” and “electroporation,” with no language restrictions applied. To maximize retrieval sensitivity and specificity, both medical subject headings (MeSH) and free-text terms were integrated into the search syntax. Supplementary studies were identified by manually screening reference lists of eligible and review articles to ensure comprehensive evidence coverage.

### 2.2 Inclusion and exclusion criteria

The inclusion criteria are as follows: (1) randomized controlled trials (RCTs) or observational clinical studies; (2) evaluation of general anesthesia, deep sedation, or conscious sedation in pulsed field ablation for atrial fibrillation; (3) reporting of comparative outcomes—such as procedural duration, fluoroscopy time, PVI success rates, and adverse event incidence—in patients undergoing PFA; (4) provision of data stratified by anesthesia type. There were no restrictions on participant inclusion based on patient race, gender, age, or the country in which the study was conducted. Excluded publication types included reviews, case reports, and animal studies.

### 2.3 Quality assessment

Two independent researchers (SW and RT) assessed the methodological quality of the included studies, with any discrepancies resolved through consensus or consultation with a third investigator. For randomized controlled trials (RCTs), quality was evaluated using the modified Jadad scale, which assesses four domains: random sequence generation, allocation concealment, blinding, and withdrawals or dropouts (16). This scale yields a maximum score of 7, where scores of 1–3 indicate low quality and 4–7 represent high quality. Observational studies were appraised using the Newcastle-Ottawa Scale (NOS) (17). The NOS evaluates study quality across three critical domains: selection of the study population, group comparability, and outcome ascertainment. A maximum of 9 points can be awarded, with scores of 0–4, 5–6, and 7–9 denoting low, moderate, and high quality, respectively.

### 2.4 Data extraction

Data extracted from the included studies encompassed the following categories: (1) study characteristics, including first author, publication year, country of origin, journal, study design, and sample size; (2) participant characteristics, such as type of atrial fibrillation, body mass index (BMI), mean age, and sex; (3) perioperative parameters and adverse events, including procedure duration, fluoroscopy time, left atrial dwell time, pulmonary vein isolation (PVI) success rate, pericardial tamponade, arteriovenous fistula, pharyngeal edema, and anesthesia-related complications. Based on the 2018 American Society of Anesthesiologists (ASA) guidelines, the CS/DS cohort in our study encompasses a spectrum of drug-induced depression of consciousness—ranging from a purposeful response to verbal commands (conscious sedation) to a purposeful response only following repeated or painful stimuli (deep sedation)—in which patients typically maintain spontaneous ventilation without requiring advanced airway interventions. In contrast, GA represents a drug-induced loss of consciousness in which patients are unarousable and frequently require advanced airway assistance.

### 2.5 Statistical analysis

Data analysis was performed using Stata software (version 18.0). Binary variables were analyzed using the risk ratio (RR), while for continuous variables, the weighted mean difference (WMD) was used. All estimates were reported with 95% confidence intervals (CIs). Statistical significance was defined as a *P* value less than 0.05. Heterogeneity was assessed using the I² statistic and Cochran's *Q* test. Significant heterogeneity was considered present if I² exceeded 50% or the *P* value was less than 0.1 (18); a sensitivity analysis was conducted to explore potential sources of heterogeneity and assess the impact of including abstract-only studies. Furthermore, univariate meta-regression analysis was performed to identify potential sources of high heterogeneity based on available continuous baseline characteristics. Publication bias was assessed using funnel plots, with symmetry evaluated visually and by Begg's and Egger's tests.

## 3 Results

### 3.1 Research results

A systematic search of databases including PubMed, Cochrane Library, and Embase was conducted, yielding an initial collection of 422 records. An additional 15 records were identified through manual searching. A total of 94 duplicate records were removed, and 75 additional records—including reviews, case reports, and other irrelevant studies—were excluded during the screening process. After evaluating the remaining 268 documents based on their abstracts or full texts in accordance with the inclusion and exclusion criteria, 9 animal studies, 204 studies with a wrong intervention or comparator, and 45 studies with incomplete or unextractable outcome data were excluded. A total of 10 studies were finally included, comprising 9 observational studies and 1 randomized controlled trial (Figure 1).

**Figure 1:**
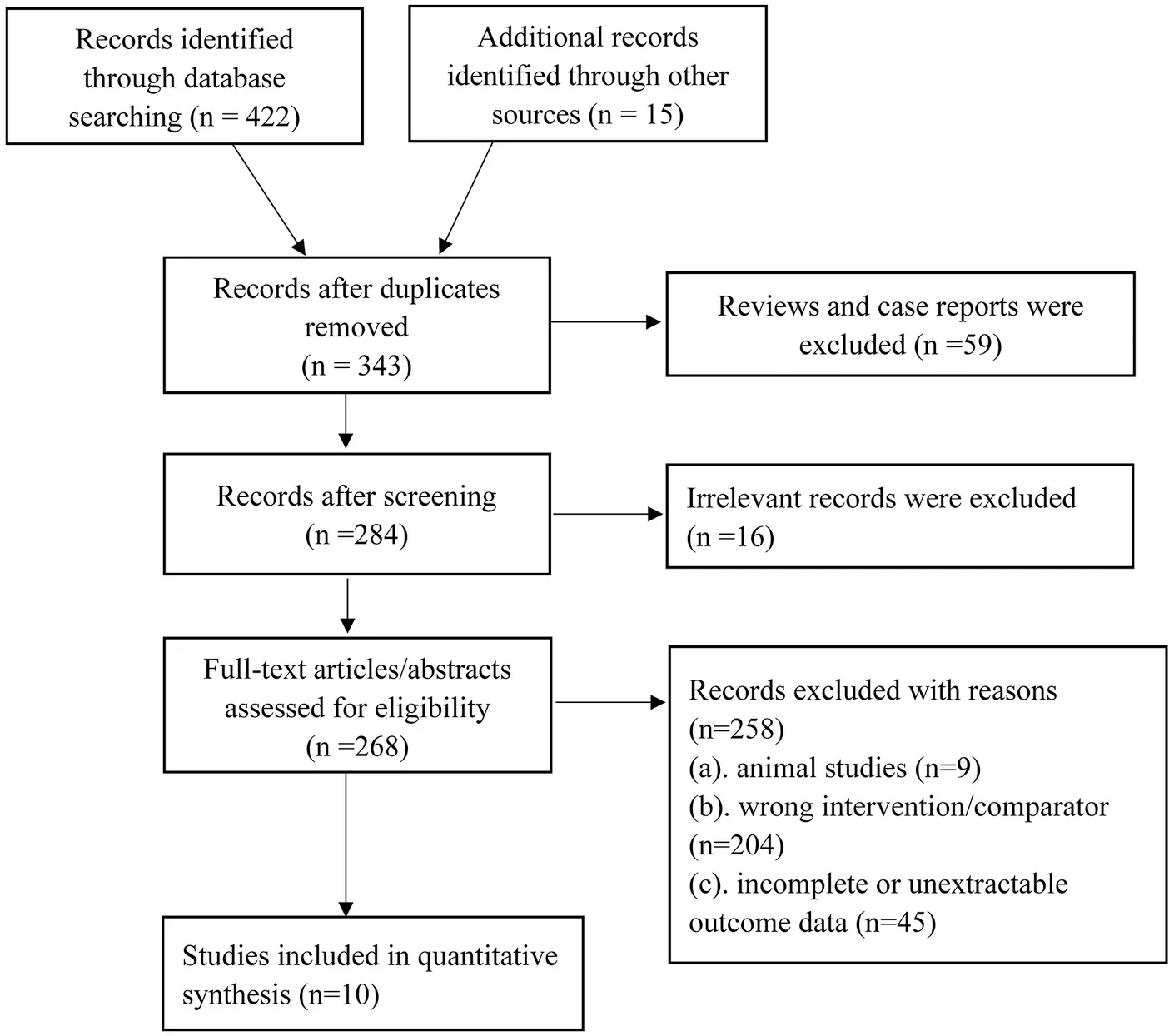
Flowchart of the study selection process.

### 3.2 Study characteristics

The ten studies included in the analysis (9, 19–27) had sample sizes ranging from 23 to 1,233, yielding a total of 3,692 patients. These studies were conducted across various countries—including Italy, Japan, Germany, the UK, the Netherlands, the Czech Republic, and Denmark—as well as in multinational, multicenter settings. The majority of participants were male. All included studies utilized pulsed field energy to achieve PVI, with additional ablation performed as clinically indicated. Regarding the types of AF covered, eight studies included both paroxysmal and persistent AF patients, one study focused exclusively on paroxysmal AF (20), and one did not explicitly specify the type of AF (19).

Anesthesia protocols varied across the included studies. Rillig et al. used a remifentanil–propofol infusion with endotracheal intubation for GA, and fentanyl–propofol for DS (21). Chun et al. utilized tracheal intubation or a laryngeal mask for GA, and propofol—often combined with midazolam, ketamine, or opioids—for DS (22). Calvert et al. used endotracheal intubation with propofol, muscle relaxants, and sevoflurane for GA, and midazolam–fentanyl boluses for mild conscious sedation with spontaneous respiration (9). Van der Graaf et al. managed GA using propofol and opioids with either a laryngeal mask or endotracheal intubation, while conscious sedation involved boluses of fentanyl, midazolam, or diazepam, supplemented with routine lidocaine (23). Sochorová et al. randomly assigned subjects to three groups: propofol–opioid DS (Group P), remimazolam–ketamine DS (Group R), and total intravenous anesthesia with propofol and sufentanil (TIVA group) (26). Alhede et al. compared three strategies: EP-led DS with propofol and fentanyl; anesthesia nurse-led DS with propofol, midazolam, and remifentanil; and anesthesiologist-led GA with endotracheal intubation (27). The anesthesia protocols for the remaining four studies were not explicitly specified, likely reflecting institutional standards of care or operator discretion.

All included studies' characteristics are summarized in Table 1. Outcomes extracted for this meta-analysis included mean procedural time, mean LA dwell time, mean total fluoroscopy time, PVI success rates, and adverse event incidence rates (categorized as major and minor). The procedural time was defined as the duration from the time the patient entered the operating room until they left at the end of the procedure.

**Table 1:** Characteristics of included study.

Study	Year	Country	Journal	Study types	Total sample size	AF type	Catheter type	Female (%)	Mean age (years)	Comparator arms	Sample size
L. Carboni et al. (19)	2025	Italy	Europace	Observational study	478	N/A	Single-shot	N/A	N/A	DS vs. GA	137 vs. 341
Shiro Nakahara et al. (20)	2025	Japan	Heart Rhythm	Observational study	24	PAF	Point-by-point	0.38	58	DS vs. GA	12 vs. 12
Andreas Rillig et al. (21)	2024	Germany	Europace	Observational study	63	PAF, PerAF	Point-by-point	0.35	64	DS vs. GA	40 vs. 23
K. R. J. Chun et al. (22)	2025	Multicenter	Europace	Observational study	1,233	PAF, PerAF	Single-shot	0.39	66	DS vs. GA	983 vs. 250
Peter Calvert et al. (9)	2025	UK	J Interv Card Electrophysiol	Observational study	23	PAF, PerAF	Single-shot	0.30	62	MCS vs. GA	8 vs. 15
M. Van der Graaf et al. (23)	2026	The Netherlands	J Interv Card Electrophysiol	Observational study	174	PAF, PerAF	Single-shot	0.33	63	CS vs. GA	42 vs. 132
R. Tilz et al. (24)	2025	Multicenter	Europace	Observational study	146	PAF, PerAF	Single-shot	0.37	64	CS/DS vs. GA	55 vs. 91
Monica Lo et al. (25)	2025	Multicenter	Heart Rhythm	Observational study	318	PAF, PerAF	Single-shot	0.36	65	CS/DS vs. GA	56 vs. 262
Veronika Sochorová et al. (26)	2025	Czech Republic	Circulation	Oxperimental study	127	PAF, PerAF	Single-shot	0.35	63	DAS1 vs. DAS2 vs. GA	42 vs. 43 vs. 42
C. Alhede et al. (27)	2025	Denmark	European Heart Journal	Observational study	1,106	PAF, PerAF	NR	0.39	63	EP-DS vs. AN-DS vs. GA	500 vs. 172 vs. 434

EP-DS, electrophysiologist-led deep sedation; AN-DS, anesthesia nurse-led deep sedation; DAS, deep analgosedation.

### 3.3 Quality assessment

This report includes a total of ten studies: nine observational studies, which were assessed for methodological quality using the NOS, and one randomized controlled trial, evaluated using the modified Jadad scale (Supplementary Table S1).

### 3.4 Baseline comparability

To ensure the validity of subsequent clinical outcome comparisons, this study rigorously assessed baseline comparability between the conscious/deep sedation and general anesthesia groups. With respect to baseline arrhythmia complexity, substantial heterogeneity was observed (*I*^2^ = 71.4%, *P* = 0.002); however, a random-effects meta-analysis of the proportion of patients with paroxysmal atrial fibrillation (AF) showed no statistically significant difference between the groups (RR = 1.00; 95% CI: 0.81–1.23; *P* = 0.987; Supplementary Figure S1A). Regarding baseline anatomical characteristics, a fixed-effects model meta-analysis of body mass index (BMI) demonstrated negligible between-group differences and confirmed baseline equivalence (WMD = −0.33 kg/m^2^; 95% CI: −0.75–0.10; *P* = 0.128; *I*^2^ = 0.0%; *P* = 0.463; Supplementary Figure S1B). Collectively, these robust findings indicate adequate baseline balance between cohorts, thereby minimizing confounding bias in the evaluation of procedural efficacy and safety outcomes.

### 3.5 Result analysis

#### 3.5.1 Mean procedural time

Nine studies reported procedural time. Substantial statistical heterogeneity was observed among the included studies (*I*^2^ = 92.6%, *P* < 0.001). Pooled results indicated that procedural time was significantly shorter in the conscious or deep sedation (CS/DS) group than in the general anesthesia (GA) group, with WMD = −7.73 (95% CI: −14.17 to −1.29; *P* = 0.019; Figure 2A). A sensitivity analysis confirmed the robustness of this finding (Supplementary Figure S2A). Univariate meta-regression revealed that baseline characteristics, including mean age (*P* = 0.193), female proportion (*P* = 0.205), and mean BMI (*P* = 0.423), were not significant sources of this high heterogeneity. Furthermore, visual inspection of the funnel plot showed approximate symmetry around the pooled effect size (Supplementary Figure S3A). Quantitative assessments using Begg's test (*P* = 0.754) and Egger's test (*P* = 0.650) indicated no significant publication bias.

**Figure 2:**
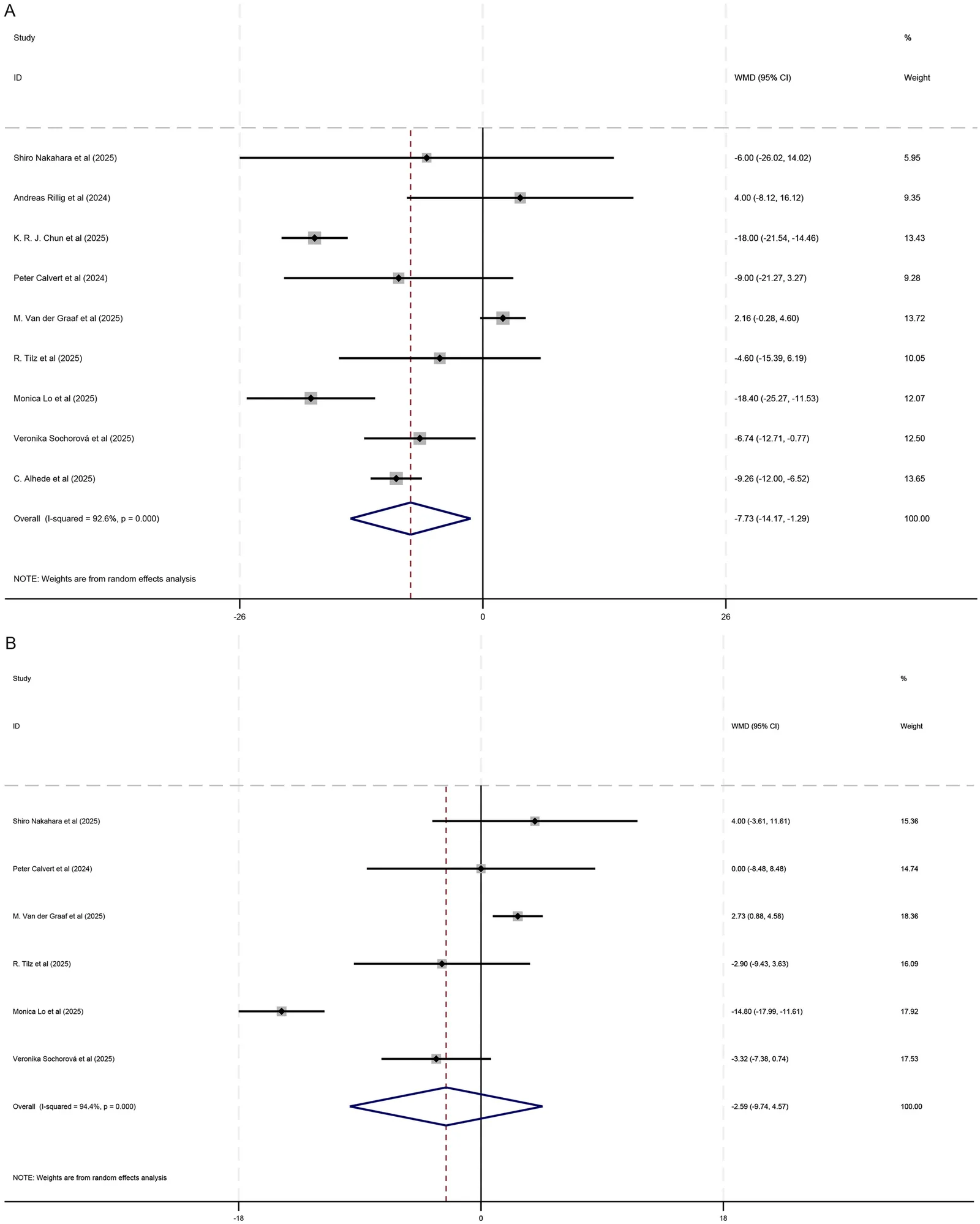

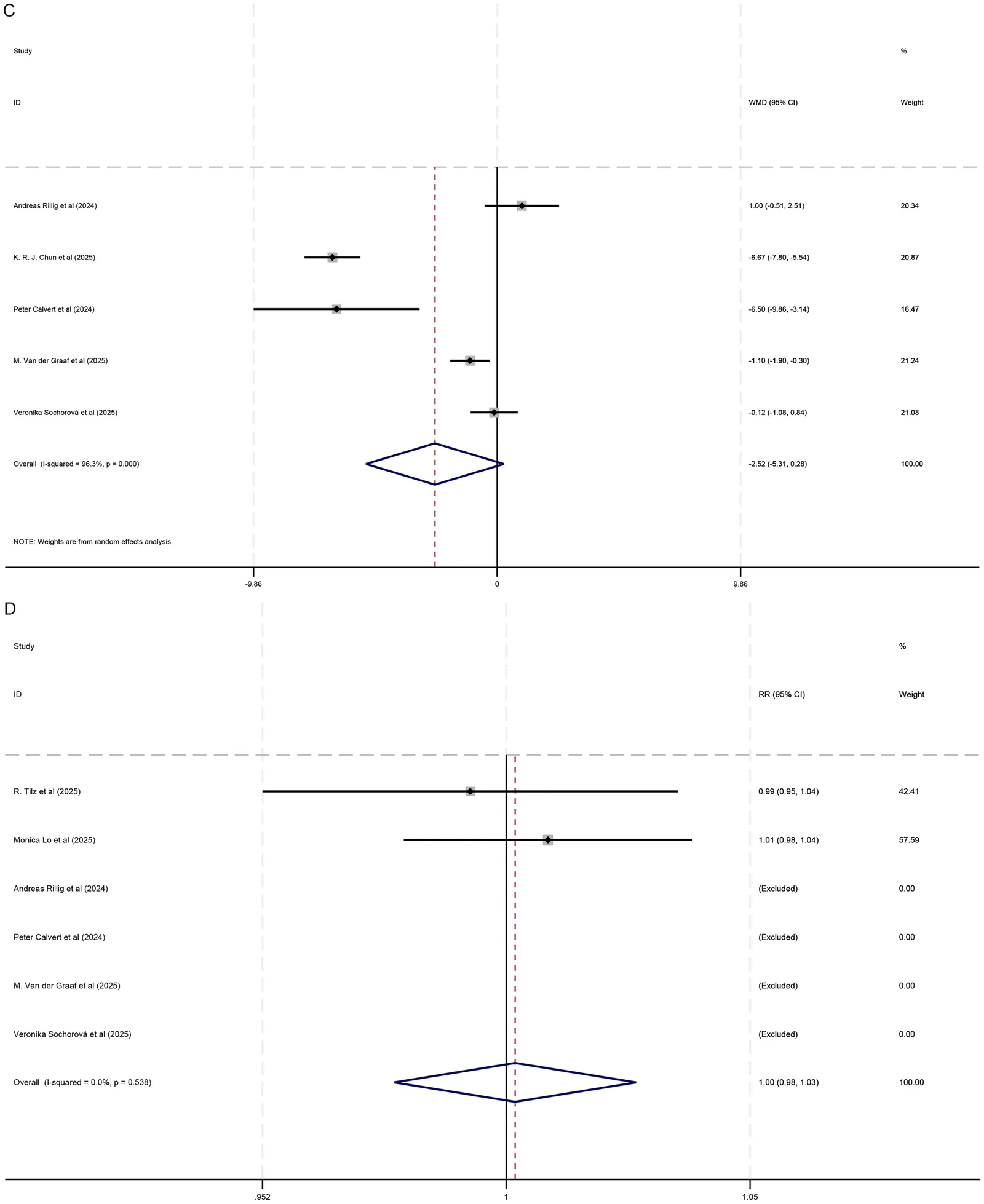

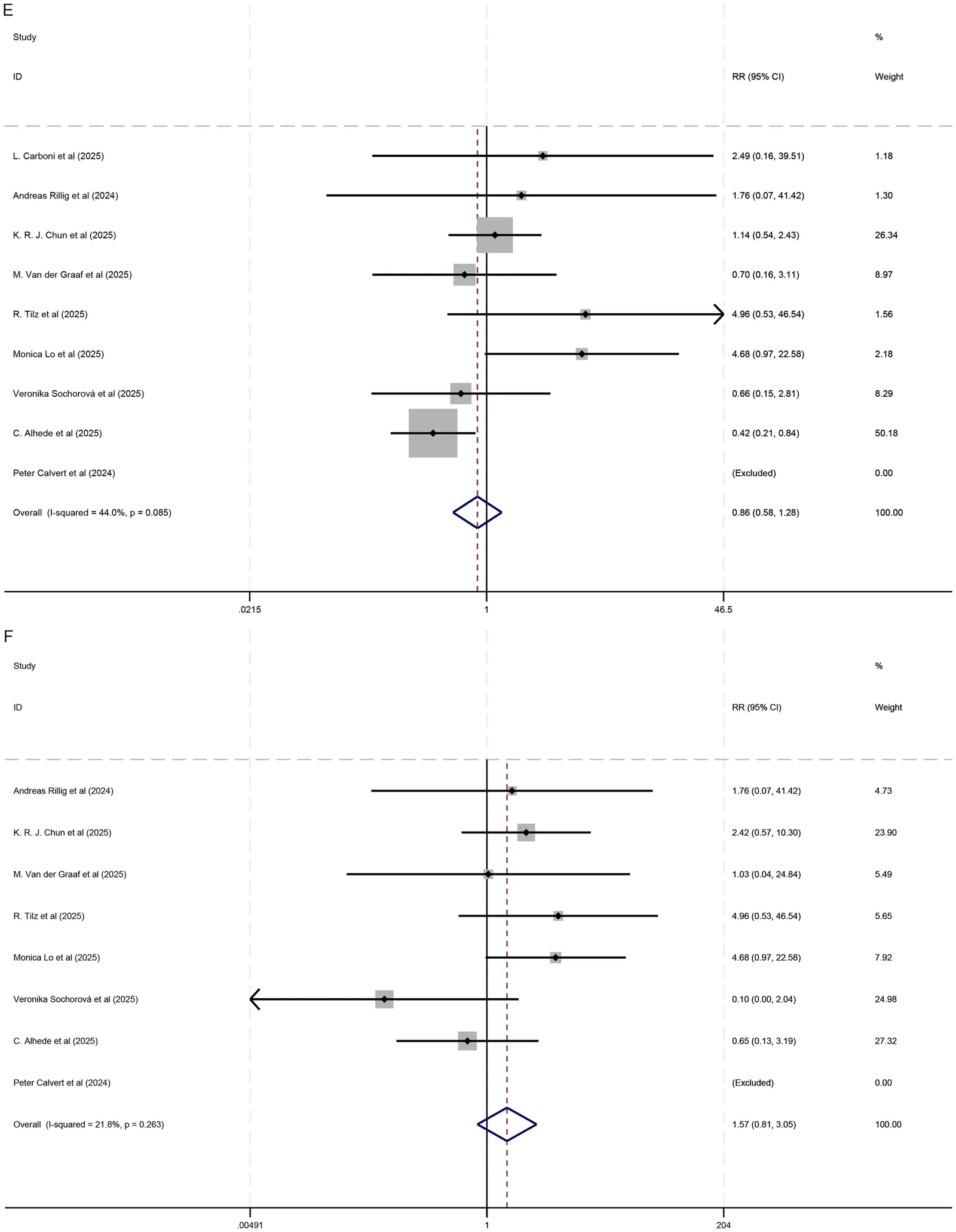

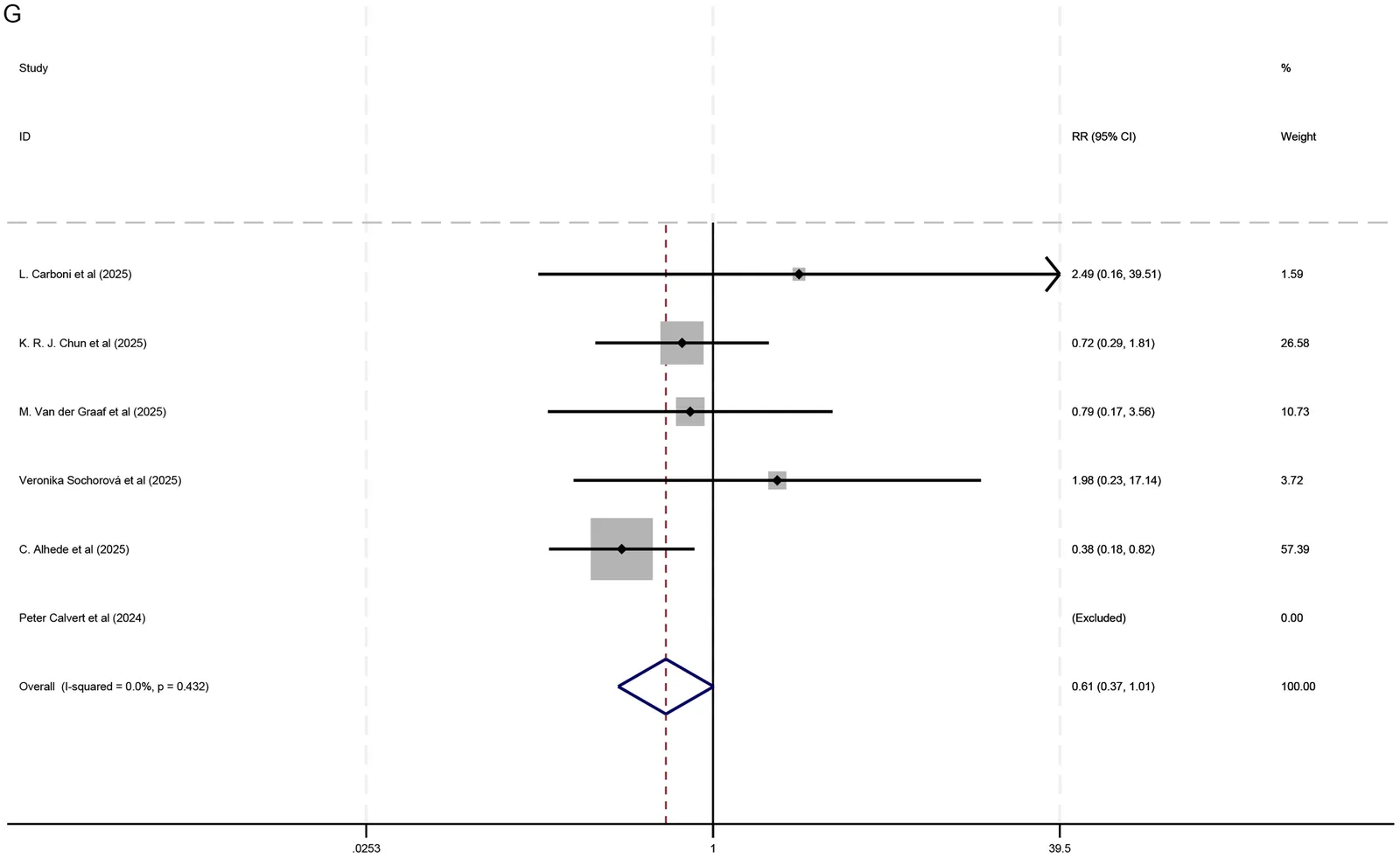
Forest plots comparing clinical outcomes between the conscious/deep sedation (CS/DS) and general anesthesia groups: **(A)** mean procedural time; **(B)** mean left atrial (LA) dwell time; **(C)** mean total fluoroscopy time; **(D)** pulmonary vein isolation (PVI) success rate; **(E)** incidence of total adverse events; **(F)** incidence of major adverse events; and **(G)** incidence of minor adverse events. For adverse events and time-based outcomes, an estimate to the left of the vertical line (RR < 1 or WMD < 0) favors CS/DS. For the PVI success rate, an estimate to the right of the vertical line (RR > 1) favors CS/DS. AF, atrial fibrillation; CI, confidence interval; RR, risk ratio; WMD, weighted mean difference.

#### 3.5.2 Mean left atrial dwell time

Six studies reported left atrial dwell time. A random-effects meta-analysis found no statistically significant difference in LA dwell time between conscious or deep sedation and general anesthesia (WMD = −2.59; 95% CI: −9.74 to 4.57; *P* = 0.479; Figure 2B). Substantial statistical heterogeneity was present across the included studies (*I*^2^ = 94.4%; *P* < 0.001). Sensitivity analysis confirmed the robustness of this null finding: exclusion of any single study did not change the overall conclusion—namely, the absence of a statistically significant effect—and the 95% confidence interval consistently included zero (Supplementary Figure S2B). Univariate meta-regression showed that mean age (*P* = 0.126), female proportion (*P* = 0.706), and mean BMI (*P* = 0.510) were not significant sources of this heterogeneity. No evidence of publication bias was observed: visual inspection of the funnel plot showed symmetry (Supplementary Figure S3B), and formal statistical tests—Begg's test (*P* = 0.707) and Egger's test (*P* = 0.670)—were nonsignificant.

#### 3.5.3 Mean fluoroscopy time

A total of five studies reported fluoroscopy time data. A random-effects meta-analysis revealed no statistically significant difference in fluoroscopy time between the conscious/deep sedation and general anesthesia groups (WMD = −2.52; 95% CI: −5.31 to 0.28; *P* = 0.077; Figure 2C). Substantial statistical heterogeneity was observed across the included studies (*I*^2^ = 96.3%; *P* < 0.001). To assess the robustness of this null finding, a leave-one-out sensitivity analysis was performed: sequential exclusion of each individual study did not meaningfully alter the pooled estimate or its statistical significance, supporting the stability of the result (Supplementary Figure S2C). Univariate meta-regression showed that mean age (*P* = 0.676), female proportion (*P* = 0.932), and mean BMI (*P* = 0.636) were not significant sources of this heterogeneity. In addition, evaluation of potential publication bias—based on visual inspection of the funnel plot (Supplementary Figure S3C] and formal statistical testing using both Begg's test (*P* = 0.806) and Egger's test (*P* = 0.665)—indicated no evidence of asymmetry or bias.

#### 3.5.4 PVI success rate

Seven studies reported the acute PVI success rate. Notably, five studies reported identical 100% success rates in both the conscious/deep sedation and general anesthesia groups; consequently, these studies provided no estimable relative risk and were excluded from the meta-analysis. The pooled RR = 1.00 (95% CI: 0.98–1.03; *P* = 0.885; Figure 2D; Supplementary Figure S3D), indicating no statistically significant difference in acute PVI success between the two anesthetic strategies. Consistent with the absence of statistical heterogeneity (*I*^2^ = 0.0%; *P* = 0.538), this null finding demonstrates high reproducibility across the analyzable evidence base.

#### 3.5.5 Incidence of adverse events

Nine studies evaluated safety outcomes in catheter ablation. One study (Calvert et al.) reported zero adverse events in both the sedation and general anesthesia groups, rendering it non-informative for relative risk estimation and thus excluded from the meta-analysis; nevertheless, its null result across treatment arms contributes to the overall evidence supporting a favorable safety profile for PFA. Adverse events were analyzed using a dual-tiered approach: first as a composite endpoint (total adverse events), and second through clinically grounded stratification—categorizing events as major or minor according to prespecified, severity-based criteria.

##### 3.5.5.1 Total adverse events

A pooled analysis of the eight included studies using a fixed-effects model revealed no statistically significant difference in the risk of adverse events following PFA between GA and CS/DS cohorts (RR = 0.86; 95% CI: 0.58–1.28; *P* = 0.457; Figure 2E). Moderate heterogeneity was observed across studies (*I*^2^ = 44.0%; *P* = 0.085). Sensitivity analysis demonstrated robustness of this finding: iterative exclusion of each individual study yielded confidence intervals that consistently included the null value (RR = 1.0; Supplementary Figure S2D), supporting result stability. In addition, assessment of small-study effects—via visual inspection of funnel plot symmetry (Supplementary Figure S3E), Begg's test (*P* = 0.386), and Egger's test (*P* = 0.112)—provided no evidence of substantial publication bias.

##### 3.5.5.2 Major adverse events

A prespecified pooled analysis of major adverse events—defined *a priori* as life-threatening or disabling complications, including cardiac tamponade, ischemic stroke, and perioperative mortality—demonstrated no statistically significant difference in risk between conscious/deep sedation and general anesthesia cohorts (RR = 1.57; 95% CI: 0.81–3.05; *P* = 0.184; Figure 2F), based on a fixed-effects model. Low heterogeneity was observed across studies (*I*^2^ = 21.8%; *P* = 0.263). Sensitivity analysis confirmed result robustness: removal of any individual study yielded a confidence interval that continued to include the null value (RR = 1.0; Supplementary Figure S2E). Finally, the funnel plot demonstrated approximate symmetry (Supplementary Figure S3F), and this absence of publication bias was statistically corroborated by both Begg's (*P* = 0.368) and Egger's tests (*P* = 0.408).

##### 3.5.5.3 Minor adverse events

A meta-analysis of five eligible studies using a fixed-effects model showed no statistically significant difference in the incidence of minor adverse events—defined as transient, non–life-threatening complications—between conscious/deep sedation and general anesthesia (RR = 0.61; 95% CI: 0.37–1.01; *P* = 0.053; Figure 2G). No heterogeneity was observed across studies (*I*^2^ = 0.0%; *P* = 0.432). Sensitivity analysis confirmed the stability of the pooled estimate: exclusion of any single study did not alter the direction of effect or statistical inference (Supplementary Figure S2F). Visual inspection of the funnel plot revealed no obvious asymmetry (Supplementary Figure S3G); Begg's test provided no evidence of asymmetry (*P* = 0.221), and Egger's test yielded a nominally significant result (*P* = 0.039). However, given the limited number of studies (*k* = 5), interpretation of the Egger test result should be cautious.

#### 3.5.6 Sensitivity analysis of abstract-only studies

A prespecified sensitivity analysis excluding studies published solely as conference abstracts did not materially alter the pooled effect sizes or statistical significance for any primary outcomes, confirming the robustness of our findings.

## 4 Discussion

Anesthesia is crucial for ensuring the safety and comfort of patients undergoing atrial fibrillation ablation. While PFA can be performed using various anesthesia regimens, the differential impact of these strategies on clinical outcomes is not yet fully understood. Our analysis systematically compared the clinical efficacy and safety of PFA under GA and CS/DS, suggesting two primary findings based on current evidence: (1) CS/DS was associated with a significant reduction in overall procedural time compared with GA, while showing no statistically significant differences in left atrial dwell time and fluoroscopy time; and (2) GA and CS/DS showed comparable safety regarding adverse events and similar acute success rates.

The reduction in total procedural time observed in the CS/DS cohort (WMD = −7.73 min) indicates a potential efficiency gain for catheterization laboratories. This temporal advantage is likely driven by circumventing the logistical burdens inherent to GA, such as prolonged induction, endotracheal intubation, and post-anesthesia recovery phases (28). For instance, a recent study utilizing a novel lattice-tip PFA catheter demonstrated that while skin-to-skin times were similar, the total laboratory occupancy time was significantly shorter under deep sedation (131 vs. 165 min) (21). Interestingly, our data yielded no statistically meaningful difference in LA dwell time or fluoroscopy time. While GA minimizes respiratory interference and involuntary movement—theoretically enhancing catheter stability—the ultra-rapid (29), millisecond-level energy delivery of PFA largely mitigates the absolute requirement for prolonged, rigid tissue contact. Consequently, the superior immobility afforded by GA might not necessarily translate into faster intra-cardiac manipulation times.

It is important to acknowledge the substantial statistical heterogeneity (*I*^2^ > 90%) observed in our meta-analysis regarding perioperative time metrics. Although our meta-regression analysis indicated that baseline patient demographics (i.e., age, sex, and BMI) did not significantly drive this heterogeneity, it likely stems from clinical and methodological variations. Specifically, the diverse array of PFA technologies—ranging from single-shot to point-by-point systems—may fundamentally influence intracardiac procedural duration. During procedures utilizing rapid single-shot systems, the ablation phase is remarkably brief, thereby reducing left atrial dwell time and increasing the relative proportion of anesthesia-related time within the overall procedural duration; consequently, the time-saving advantage of CS/DS over GA during anesthesia induction and recovery becomes proportionally more prominent. Conversely, when point-by-point systems are employed—which necessitate prolonged catheter stability and extended left atrial dwell time—or when centers are early in their learning curve, such prolonged intracardiac manipulation can attenuate this relative time benefit, with variable effects on fluoroscopy use. Moreover, highly variable and non-standardized pharmacologic regimens across centers can introduce unpredictable fluctuations in anesthesia induction and recovery times. Ultimately, the confluence of these device-related, experiential, and pharmacologic variations potentially contributes to the observed high statistical heterogeneity, limiting the capacity to fully account for it in pooled analyses.

In parallel with these efficiency gains, our pooled analysis indicates consistently high acute PVI success rates in both the CS/DS and GA cohorts. This robust efficacy may be largely attributable to the near-instantaneous nature of PFA (30), which potentially renders lesion formation less susceptible to the respiratory fluctuations and transient patient movements associated with CS/DS. Although general anesthesia has historically been preferred to enhance catheter stability and mapping fidelity, recent studies suggest that contemporary 3D electroanatomical mapping—when integrated with carefully titrated deep sedation—is sufficient to support stable map acquisition and successful PVI (31–33). Consequently, while general anesthesia remains a valuable option for high-risk or anatomically complex cases, CS/DS represents a viable, evidence-supported alternative for routine ablation procedures, achieving comparable acute clinical success without the logistical and physiological burdens associated with general anesthesia.

The safety profile of CS/DS appears comparable to that of GA, with no statistically significant difference in the incidence of major complications. Notably, a borderline statistical trend (*P* = 0.053) suggests a potentially lower incidence of minor adverse events in the CS/DS group—a finding that may be plausibly explained by avoidance of endotracheal intubation–related airway trauma and attenuation of profound hemodynamic fluctuations commonly associated with deep GA (34, 35). While our analysis evaluated the overall safety of CS/DS, current literature provides important context, indicating that the safety and efficacy of CS/DS are critically dependent on the specific pharmacological regimen. For example, prior studies suggest that standard mild conscious sedation—typically comprising bolus administration of midazolam and fentanyl—often provides suboptimal analgesia, leading to increased intraoperative pain perception, reduced patient cooperation, and unplanned escalation of sedative dosing (36, 37). Conventional bolus-based propofol deep sedation, though widely used, was associated with a notably high periprocedural hypoxemia rate of 85.7% in the COOPERATIVE-PFA randomized controlled trial. By comparison, optimized continuous infusion protocols—such as remimazolam co-administered with low-dose ketamine—have been reported to achieve a favorable balance: they facilitate effective analgesia and hemodynamic stability while consistently preserving spontaneous ventilation, thereby helping to reduce the risk of respiratory and cardiovascular adverse events (38, 39). These contextual findings suggest that although avoiding GA may offer substantial clinical benefits, the full safety advantage of CS/DS might be optimally achieved through rigorous, evidence-based regimen selection and individualized, titrated delivery of sedative–analgesic agents. Given the significant variations in drug selection and the frequent omission of precise dosage and administration details across the included studies (Table 2), a granular, agent-specific subgroup analysis was not feasible in this study. Therefore, future prospective trials with fully standardized and comprehensively reported sedation protocols are warranted to fill this evidence gap.

**Table 2:** Details of anesthesia and sedation protocols.

Study	General anesthesia (GA) protocol	Conscious/deep sedation (CS/DS) protocol
L. Carboni et al. (19)	NR	NR
Shiro Nakahara et al. (20)	NR	NR
Andreas Rillig et al. (21)	Propofol (1% solution dose NR, bolus + continuous infusion); Remifentanil (NR, continuous infusion); Succinylcholine/Rocuronium (NR, bolus); Neostigmine/Sugammadex (NR, NR)	Fentanyl (25 µg initial + 25 µg before ablation, bolus); Propofol (1% solution 3–5 mg initial 5 mg/kg/h maintenance, bolus + continuous infusion)
K. R. J. Chun et al. (22)	NR	Propofol (NR, NR); Ketamine (NR, NR); Midazolam (NR, NR); Oxycodone (NR, NR); Remifentanil (NR, NR); Fentanyl (NR, NR)
Peter Calvert et al. (9)	Propofol (1–2 mg/kg, bolus); Muscle relaxant (NR, NR); Sevoflurane (NR, inhalation maintenance)	Midazolam (1–2 mg initial/mean total 5.12 mg, bolus); Fentanyl (50 mcg initial/mean total 209 mcg, bolus); Paracetamol (NR, intravenous)
M. Van der Graaf et al. (23)	Propofol (NR, bolus + continuous infusion); Opioid (NR, continuous infusion); Rocuronium (NR, bolus); Fentanyl (NR, bolus); Atropine (0.5 mg, bolus)	Fentanyl (50 mcg, bolus); Diazepam (5 mg, bolus) OR Midazolam (2.5 mg, bolus); Atropine (0.5 mg, bolus); Lidocaine (1 mg/kg, bolus); Etomidate (5 mg max 20 mg, bolus)
R. Tilz et al. (24)	NR	NR
Monica Lo et al. (25)	NR	NR
Veronika Sochorová et al. (26)	Propofol (5–8 µg/mL induction 3–6 µg/mL maintenance, target-controlled continuous infusion); Rocuronium (0.2–0.4 mg/kg, bolus); Sufentanil (NR, bolus)	Arm P: Propofol (0.8–1.0 mg/kg loading + 0.5 mg/kg boluses, intermittent boluses); Midazolam (NR, intermittent boluses); Sufentanil (5–10 µg, intermittent boluses)
		Arm R: Remimazolam (0.5 mg/kg/h continuous + 2.5 mg boluses, continuous infusion + boluses); Ketamine (1 mg/kg initial + 0.5 mg/kg boluses, boluses); Sufentanil (NR, bolus)
C. Alhede et al. (27)	NR	EP-DS: Propofol (NR, NR); Fentanyl (NR, NR)
		AN-DS: Propofol (NR, NR); Midazolam (NR, NR); Remifentanil (NR, NR)

Data are reported in the following format: Drug Name (Dose, Route of Administration).

GA, General Anesthesia; CS/DS, Conscious Sedation/Deep Sedation; NR, Not Reported; Arm P, Propofol arm; Arm R, Remimazolam arm; EP-DS, electrophysiologist-led deep sedation; AN-DS, anesthesia nurse-led deep sedation.

Beyond intraoperative performance metrics, the clinical viability of CS/DS aligns well with the clinical demand for enhanced postprocedural recovery. From a clinical standpoint, avoiding GA eliminates airway trauma associated with endotracheal intubation and mechanical ventilation, thereby potentially accelerating postprocedural recovery. This can translate into shorter post-procedural ward recovery times and a reduced overall hospital length of stay (LOS), facilitating the earlier initiation of ambulation (40). Importantly, from a patient-centered perspective, CS/DS is associated with a reduced incidence of postoperative nausea and vomiting (PONV)—a common adverse effect of deep inhalational or intravenous anesthetic agents—which may meaningfully contribute to improved patient-reported outcomes and overall satisfaction (41).

Critically, these findings may carry important implications for healthcare resource allocation and procedural workflow efficiency. In numerous healthcare systems worldwide, access to dedicated anesthesiology support remains a key operational constraint, often prolonging room turnover times and limiting laboratory capacity (42). High-quality registry evidence demonstrates that protocol-driven, titrated deep sedation—safely administered by credentialed electrophysiology personnel or specialized anesthesia nursing teams—is both clinically feasible and associated with an excellent safety profile (43, 44). Recognition of CS/DS as a robust, evidence-based alternative to GA has the potential not only to lower overall hospitalization costs but also to enhance procedural throughput and resource utilization (7). Ultimately, this facilitates the broader adoption of PFA technology—supporting scalable and sustainable implementation across diverse practice settings, including resource-limited environments and high-volume centers—while appearing to maintain comparable acute procedural efficacy and patient safety.

## 5 Limitations

This study has several limitations. First, the diverse PFA techniques across studies—such as varying catheter designs and energy parameters—inevitably introduce some clinical heterogeneity. Our results therefore reflect the overall class effect of PFA; device-specific nuances warrant further dedicated study. Second, the current literature lacks direct comparisons of the effects of different anesthesia methods in PFA from a patient-centered perspective, limiting the use of patient-reported outcomes in clinical decisions. Third, as the majority of the included studies are non-randomized observational cohorts, our findings are inherently susceptible to selection bias and confounding by indication. Although baseline comparability was observed in variables such as BMI and atrial fibrillation type, unmeasured clinical confounders—including baseline frailty, procedural complexity, and institutional practice patterns—cannot be entirely ruled out. Consequently, the observed differences in perioperative efficiency and safety outcomes should be interpreted with caution, as they may be partially influenced by the selective assignment of anesthesia modalities in routine clinical practice. Fourth, this study lacks comparative data on long-term outcomes; larger-scale studies with extended follow-up periods are required to validate these findings. Fifth, the limited number of studies and the inconsistent reporting of sedation depth across the original trials precluded a robust, separate subgroup analysis (GA vs. CS and GA vs. DS).

## 6 Conclusion

In this meta-analysis evaluating anesthesia strategies for atrial fibrillation ablation using PFA, CS/DS was associated with reduced overall procedural time compared with GA, while acute PVI success rates and safety profiles were comparable. The wide confidence intervals and borderline statistical trends observed for left atrial dwell time and fluoroscopy time necessitate cautious interpretation. However, given the limited number of included studies and relatively small sample sizes, these findings should be considered preliminary. Future high-quality, large-scale randomized controlled trials are needed to confirm these results and further optimize standardized sedation protocols.

## Data Availability

The raw data supporting the conclusions of this article will be made available by the authors, without undue reservation.
