# Supplementary material for: Efficacy and safety of conscious/deep sedation versus general anesthesia in pulsed field ablation for atrial fibrillation: a systematic review and meta-analysis
Source: Front Cardiovasc Med. 2026 Oct 23;13:1933701. doi: 10.3389/fcvm.2026.1933701 (PMC13741799; doi:10.3389/fcvm.2026.1933701)
Supplement: Supplementary file 1 [file Datasheet1.docx]

**Efficacy and safety of conscious/deep sedation versus general anesthesia in pulsed field ablation for atrial fibrillation: A systematic review and meta-analysis**

**Supplementary material**

**Table of content**

**Supplementary table 1.** Quality assessment of studies……………………………………1

**Supplementary figure S1.** Forest plots for baseline comparisons…………………...2

**Supplementary figure S2.** Leave-one-out sensitivity analyses………………………………………...……………………………………...4

**Supplementary figure S3.** Funnel plots for the assessment of publication bias…………………………………………………...………………………...……...7

**Supplementary table 1.** Quality assessment of studies

| Study | Year | Study types | Score |
| --- | --- | --- | --- |
| L. Carboni et al | 2025 | observational study | 7(NOS) |
| Shiro Nakahara et al | 2025 | observational study | 6(NOS) |
| Andreas Rillig et al | 2024 | observational study | 8(NOS) |
| K. R. J. Chun et al | 2025 | observational study | 8(NOS) |
| Peter Calvert et al | 2024 | observational study | 7(NOS) |
| M. Van der Graaf et al | 2025 | observational study | 7(NOS) |
| R. Tilz et al | 2025 | observational study | 7(NOS) |
| Monica Lo et al | 2025 | observational study | 7(NOS) |
| Veronika Sochorová et al | 2025 | randomized controlled trial | 5(Jadad) |
| C. Alhede et al | 2025 | observational study | 7(NOS) |

**Supplementary figure S1.** Forest plots for baseline comparisons

(A) Comparison of PAF proportions

(B) Comparison of BMI
**Supplementary Figure S2.** Leave-one-out sensitivity analyses

(A) mean procedural time

(B) mean left atrial (LA) dwell time

(C) mean total fluoroscopy time

(D) incidence of total adverse events

(E) incidence of major adverse events

(F) incidence of minor adverse events

**Supplementary figure S3.** Funnel plots for the assessment of publication bias

(A) mean procedural time

(B) mean left atrial (LA) dwell time

(C) mean total fluoroscopy time

(D) pulmonary vein isolation (PVI) success rate

(E) incidence of total adverse events

(F) incidence of major adverse events

(G) incidence of minor adverse events
